# Supplementary material for: Behavioural correlations of the domestication syndrome are decoupled in modern dog breeds
Source: Nat Commun. 2019 Jun 3;10:2422. doi: 10.1038/s41467-019-10426-3 (PMC6546797; doi:10.1038/s41467-019-10426-3)
Supplement: Supplementary file 1 — Supplementary Information [file 41467_2019_10426_MOESM1_ESM.pdf]

- Supplementary Information -

Behavioural correlations of the domestication syndrome are decoupled in modern dog breeds

Hansen Wheat et al.

**Supplementary Table 1.** Behavioural assays	2
**Supplementary Table 2.** Summary statistics	3
**Supplementary Table 3.** Assessment of confounding factors	4
**Supplementary Table 4.** Full model on 16 individual behavioural correlations	5
**Supplementary Table 5.** Correlation matrix based on breed means	6
**Supplementary Figure 1.** Effect sizes of behavioural correlations	7
**Supplementary Figure 2.** Funnel plots for sociability correlations	8
**Supplementary Figure 3.** Funnel plots for playfulness correlations	9
**Supplementary Figure 4.** Funnel plots for fearfulness correlations	10
**Supplementary References**	10

Supplementary Table 1. Behavioural assays. Description of the behavioural assays in the DMA test and the elements in each behavioural category. For full description of the DMA test battery see Svartberg and Forkman (2002)[1].

Behaviour	Description, context and scoring
Sociability	The dog is on leash led by its handler (a familiar person, usually the owner). The test leader, a stranger, approaches and greets the dog and handler. The test leader takes the leash and goes for a short walk with the dog, which includes a standstill during which the test leader pets the dog. Returning the dog to the handler, the test leader performs a brief physical examination of the dog. The dog greeting reaction and reaction to the physical examination is scored on a scale from 1 (rejecting contact with growling and/or bite attempt) to 5 (escalated contact initiative, i.e. jumping, whimpering, barking etc.).
Playfulness	The dog is taken off the leash and the handler and the test leader start throwing a rag between them. The rag is thrown for a distance, and if the dog runs to fetch it, the test leader calls the dog back. The test leader then invites the dog to play tug of war. The dog is scored based on its interest, engagement and activity in playing on a scale from 1 (no interest, no playing) to 5 (fast engagement, very active in playing).
Aggression (distant threat)	A person dressed in a hooded cape appears from a hiding spot 40 meters away from the dog and the handler. The person is clapping his hands and moving sideways. After having moved 3 meters sideway, the person stops while widening the cape and crouching at the same time. This sequence is repeated three times while moving altogether 12 meters sideway, and then hiding again. The dog's aggressive response towards the person in the cape is scored while it is held next to its handler on a scale from 1 (no signs of aggression or threat display) to 5 (growling, snarling, raised hackles, raised tail etc.).
Fearfulness (sudden threat)	The handler is walking the dog on a leash when a human-like dummy is suddenly pulled up in front of the dog from a distance of two meters. The handler releases the dog upon appearance of the dummy. The dog's immediate response to the dummy is scored on a scale from 1 (no hesitation or only a brief stop) to 5 (fleeing more than 5 meters).
Aggression (sudden threat)	The context is the same as in the test for Fearfulness – sudden threat and the behavioural assessment follows immediately after the fear response has been assessed. The dog's aggressive behaviour towards the dummy is scored on a scale from 1 (no threatening or aggressive behaviour) to 5 (display of threatening behaviour, attacking, possibly biting the dummy).
Fearfulness (persistent threat)	The handler is standing still with the dog on a leash when two people dressed in white sheets with white hoods over their heads slowly approach the dog from 20 meters distance. The ghosts are 25 meters apart when starting the approach, creating a triangular shape with the dog. After three minutes the ghosts close in on the dog and the handler then releases the dog. The dog is scored based on the immediate behaviour towards the ghosts on a scale from 1 (stays in place in front or next to the handler) to 5 (attempting to move back further than the length of the leash and/or attempting to leave the scene. Possibly trying to flee).
Aggression (persistent threat)	The context is the same as in the test for Fearfulness – persistent threat and the behavioural assessment follows immediately after the fear response has been assessed, while the ghosts are approaching the dog. The dog's aggressive behaviour towards the dummy is scored on a scale from 1 (no threatening or aggressive behaviour) to 5 (display of threatening behaviour, several attemps of attacking the ghosts).

Supplementary Table 2. Summary statistics. Sample sizes (N = individual dogs in each breed category) and percentage are given for both males and females, as well as mean age (± standard error) upon testing and testing location for ancient and modern breeds. Each of the three geographic testing locaions (East, South and North) were divided into the categories City or Rural. The three geographical categories were based on the three regions of Sweden classified by the European Union and city-locations were assigned to owners living witin the city limit of the three large cities in Sweden (based on populations: Stockholm, Göteborg and Malmö). Dogs living outside the three cities were classified as rural. None of the three largest cities are located in the northern part of Sweden, and all dogs from this geographical area were therefore given the category rural. For the analyses the three geographic locations and the city or rural categories were combined to give five different combinations of locations in total.

Parameter	Ancient	Modern
N	251	75,907
Male	130 (52%)	36,978 (49%)
Female	121 (48%)	38,929 (51%)
Mean age (days ± SE)	709.5 (± 16.3)	599.3 (± 0.8)
Testing location:		
East - City	30 (12%)	11,149 (15%)
East - Rural	44 (18%)	10,402 (14%)
South - City	3 (1%)	1,705 (2%)
South - Rural	120 (48%)	35,560 (47%)
North - Rural	54 (22%)	17,091 (23%)

Supplementary Table 3. Assessment of confounding factors. Linear mixed effects models assessing how each behaviour in our analyses is associated with the potentially confoundnig variables breed category (ancient and modern), sex, age in days and testing location. Observer ID and breed were included as random effects. Non-significant interaction terms were removed and simplified models are presented.

Behaviour	Predictor	χ^2	df	p
Sociability	Breed category	0.03	1	0.86
	Sex	363.21	1	<0.001
	Age	454.01	1	<0.001
	Testing location	4.67	2	0.10
Playfulness	Breed category	5.86	1	0.02
	Sex	570.39	1	<0.001
	Age	946.49	1	<0.001
	Testing location	1.55	2	0.46
Aggression (distant)	Breed category	0.07	1	0.78
	Sex	156.38	1	<0.001
	Age	110.55	1	<0.001
	Testing location	19.45	2	<0.001
Fear (sudden)	Breed category	0.59	1	0.44
	Sex	760.96	1	<0.001
	Age	332.22	1	<0.001
	Testing location	24.60	2	<0.001
Aggression (sudden)	Breed category	3.10	1	0.08
	Sex	54.72	1	<0.001
	Age	142.44	1	<0.001
	Testing location	10.70	2	0.005
Aggression (persistent)	Breed category	9.53	1	0.002
	Sex	0.26	1	0.61
	Age	162.26	1	<0.001
	Testing location	20.31	2	<0.001
Fear (persistent)	Breed category	0.03	1	0.87
	Sex	527.51	1	<0.001
	Age	404.21	1	<0.001
	Testing location	18.51	2	<0.001

Supplementary Table 4. Full model on 16 individual behavioural correlations. Full Bayesian general linear mixed model on Z_r values based on Pearson correlations between sociability (Soc), playfulness (play), fearfulness - sudden threat (FearS), fearfulness - persistent threat (FearP), aggression - distant threat(AggrD), aggression - sudden threat (AggrS) and aggression - persistent threat (AggrP) in ancient and modern breeds (Breed type). Expected correlation direction (Exp. cor) is given as positive or negative. Post mean Z_r values and corresponding 95% confidence intervals are given for each behavioural correlation. Effective sample size is N = 1000 for all runs. Note that the model for one of the expected negative correlations (sociability vs. aggression (P)) did not converge and was therefore not included.

Correlation	Exp. cor.	Predictor	Post mean	$95\%CI_{low}$	$95\%CI_{up}$
Soc v. Play	Positive	Intercept	0.308	0.149	0.449
		Breed type	-0.031	-0.173	0.132
FearS v. AggrD	Positive	Intercept	0.094	-0.040	0.224
		Breed type	-0.026	-0.161	0.105
FearS v. AggrS	Positive	Intercept	0.132	-0.003	0.306
		Breed type	-0.063	-0.228	0.076
FearS v. AggrP	Positive	Intercept	0.019	-0.127	0.158
		Breed type	-0.033	-0.182	0.104
FearP v. AggrD	Positive	Intercept	-0.008	-0.143	0.123
		Breed type	0.048	-0.083	0.185
FearP v. AggrS	Positive	Intercept	-0.070	-0.208	0.066
		Breed type	0.045	-0.088	0.185
FearP v. AggrP	Positive	Intercept	0.261	0.109	0.393
		Breed type	-0.079	-0.216	0.065
Soc v. AggrD	Negative	Intercept	-0.136	-0.263	-0.013
		Breed type	0.093	-0.033	0.221
Soc v. AggrS	Negative	Intercept	-0.109	-0.269	-0.013
		Breed type	0.095	-0.006	0.253
Soc v. FearS	Negative	Intercept	-0.220	-0.354	-0.088
		Breed type	0.141	0.002	0.271
Soc v. FearP	Negative	Intercept	-0.061	-0.200	0.074
		Breed type	0.021	-0.112	0.163
Play v. AggrD	Negative	Intercept	-0.043	-0.167	0.084
		Breed type	0.040	-0.084	0.169
Play v. AggrS	Negative	Intercept	-0.047	-0.190	0.078
		Breed type	0.093	-0.052	0.218
Play v. AggrP	Negative	Intercept	1.033	-0.048	0.233
		Breed type	-1.003	-0.203	0.081
Play v. FearS	Negative	Intercept	-0.150	-0.288	-0.027
		Breed type	0.086	-0.030	0.232
Play v. FearP	Negative	Intercept	-0.134	-0.283	-0.009
		Breed type	0.101	-0.026	0.250

Supplementary Table 5. Correlation matrix for based on breed means. Correlation coefficients (**r**) from Pearson correlations are presented for each pairwise behavioural combination (Sociability (Soc), Playfulness (Play), Aggression – distant threat (AggrD), Fearfulness – sudden threat (FearS), Aggression – sudden threat (AggrS), Fearfulness – persistent threat (FearP) and Aggression – persistent threat (AggrP) for ancient (below the diagonal) and modern (above the diagonal) dog breeds. The a priori direction of correlations between the behaviours based on the domestication syndrome hypothesis is presented in brackets, with (+) indicating expected positive correlations and (-) indicating expected negative correlations. We did not consider correlations using alternative measures of the same behavior (e.g. correlations between alternative measures of fear or aggression) as the domestication syndrome hypothesis does not make explicit predictions about within behaviour correlations; these cases are marked with NA in the table. Bold values indicate cases where the correlation coefficients were exaggerated in the direction-specific manner predicted by the domestication syndrome hypothesis. Note that of the 17 behavioural correlations, 15 are exaggerated in ancient breeds, one is exaggerated in modern breeds, and one goes in the opposite direction from the predictions of the domestication syndrome hypothesis for both ancient and modern breeds.

	Soc	Play	AggrD	FearS	AggrS	AggrP	FearP
Soc		0.37 (+)	0.02 (-)	-0.30 (-)	0.37 (-)	0.11 (-)	-0.44 (-)
Play	0.66 (+)		-0.17 (-)	-0.18 (-)	0.31 (-)	0.18 (-)	-0.45 (-)
AggrD	-0.16 (-)	-0.74 (-)		0.21 (+)	NA	NA	0.21 (+)
FearS	-0.15 (-)	-0.72 (-)	0.58 (+)		-0.62 (+)	-0.68 (+)	NA
AggrS	0.41 (-)	-0.18 (-)	NA	0.36 (+)		NA	-0.24 (+)
AggrP	-0.23 (-)	-0.74 (-)	NA	0.55 (+)	NA		-0.04 (+)
FearP	-0.73 (-)	-0.79 (-)	0.40 (+)	NA	-0.11 (+)	0.66 (+)	

Supplementary Figure 1. Effect sizes of 16 behavioural correlations. Mean effect sizes (Z_r) and 95% confidence intervals for 16 individual behavioural correlations between Sociability, Playfulness, Fearfulness - sudden threat (Fear (S)), Fearfulness - persistent threat (Fear (P)), Aggression - distant threat (Aggression (D)), Aggression - sudden threat Aggression (S)) and Aggression - persistent threat (Aggression (P)). Correlations are divided into expected positive (top seven) and negative (bottom nine) correlations. Note that the model for one of the expected negative correlations (sociability vs. aggression (P)) did not converge and was therefore not included. Source data are available as a Source Data file.

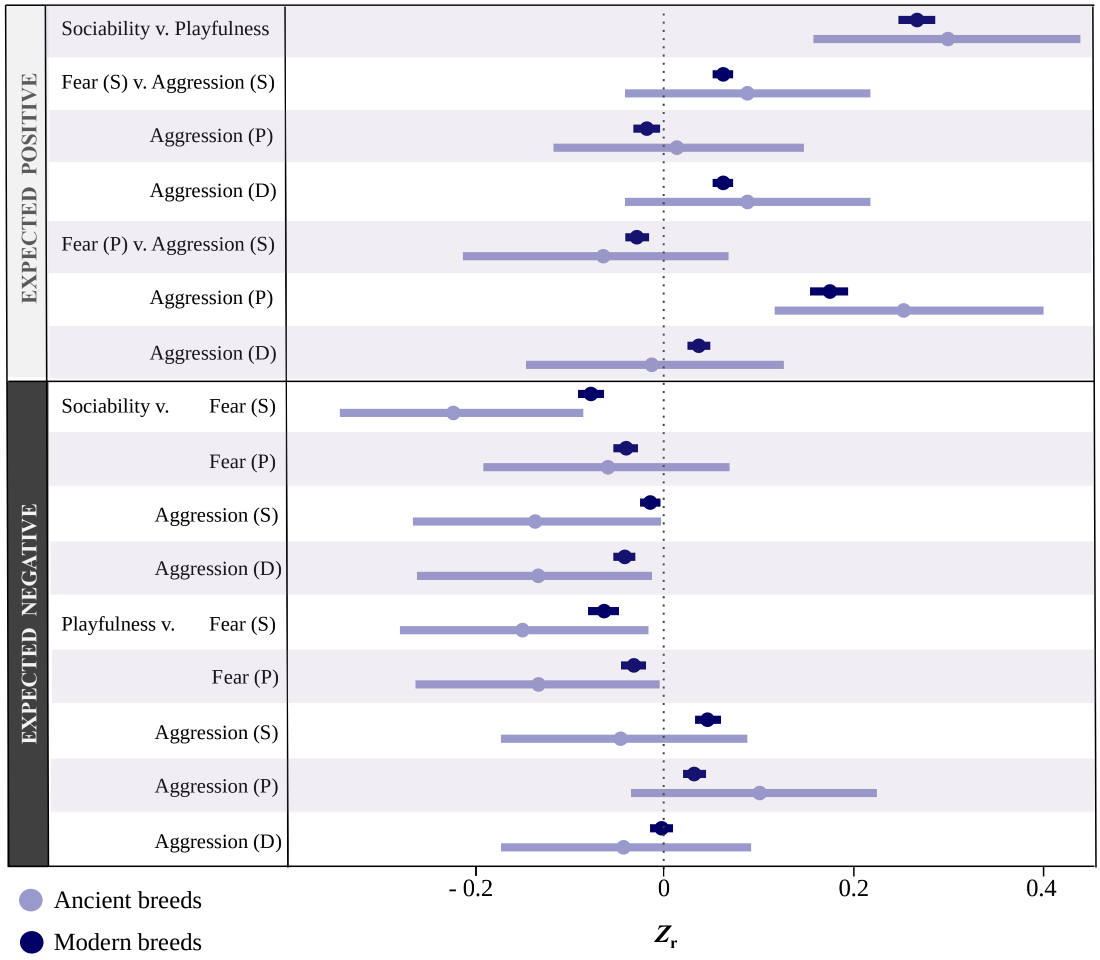

Supplementary Figure 2. Funnel plots for sociability correlations. Funnel plots for the correlations between sociability and playfulness, fearfulness - sudden threat (Fear S) and fearfulness - persistent threat (Fear (P)), aggression - distant threat (Aggression (D)), aggression - sudden threat (Aggression (S)) and aggression – persistent threat (Aggression (P)) for all 78 dog breeds. The model on the correlation between Sociablity and Aggression - persistent threat (Aggression (P)) did not converge and was not included in the result section of the manuscript. Estimated mean (red line) for a given correlation overlaps completely with the fitted regression mean (blue line). N = number of dogs within each of the 78 breeds in our study. Source data are available as a Source Data file.

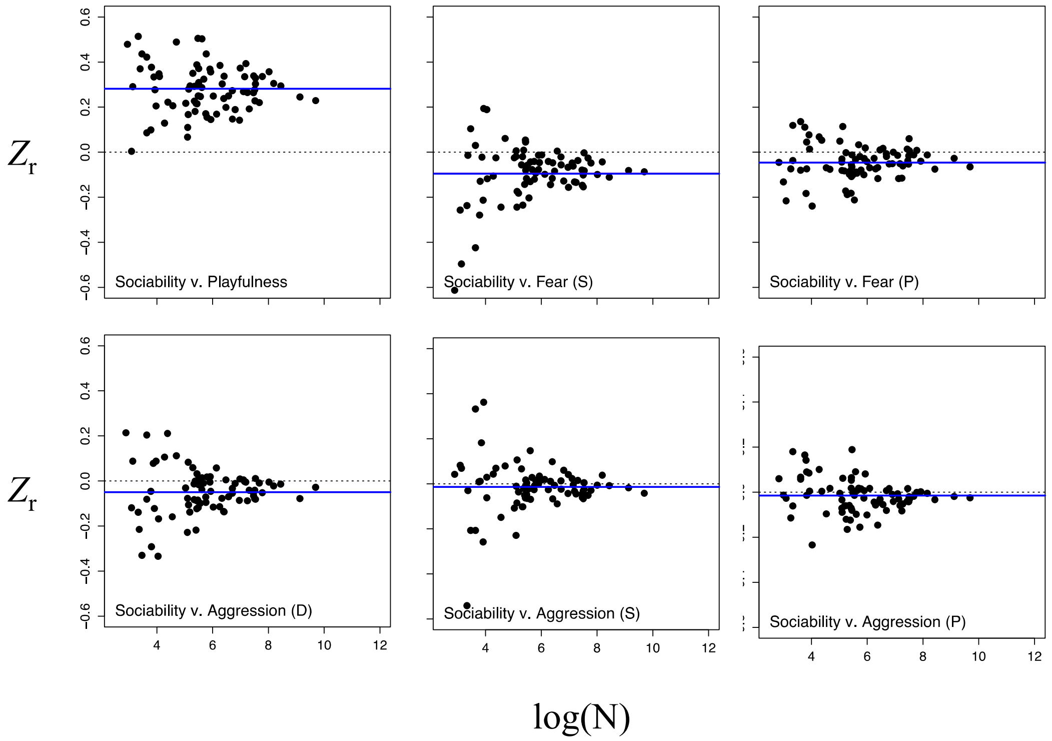

Supplementary Figure 3. Funnel plots for playfulness correlations. Funnel plots for the correlations between Playfulness and Aggression - distant threat (Aggression (D)), Aggression - sudden threat (Aggression (S)), Aggression - persistent threat (Aggression (P)), Fearfulness - sudden threat (Fear (S)) and fearfulness - persistent threat (Fear (P)) for all 78 dog breeds. Estimated mean (red line) for a given correlation overlaps completely with the fitted regression mean (blue line). N = number of dogs within each of the 78 breeds in our study. Source data are available as a Source Data file.

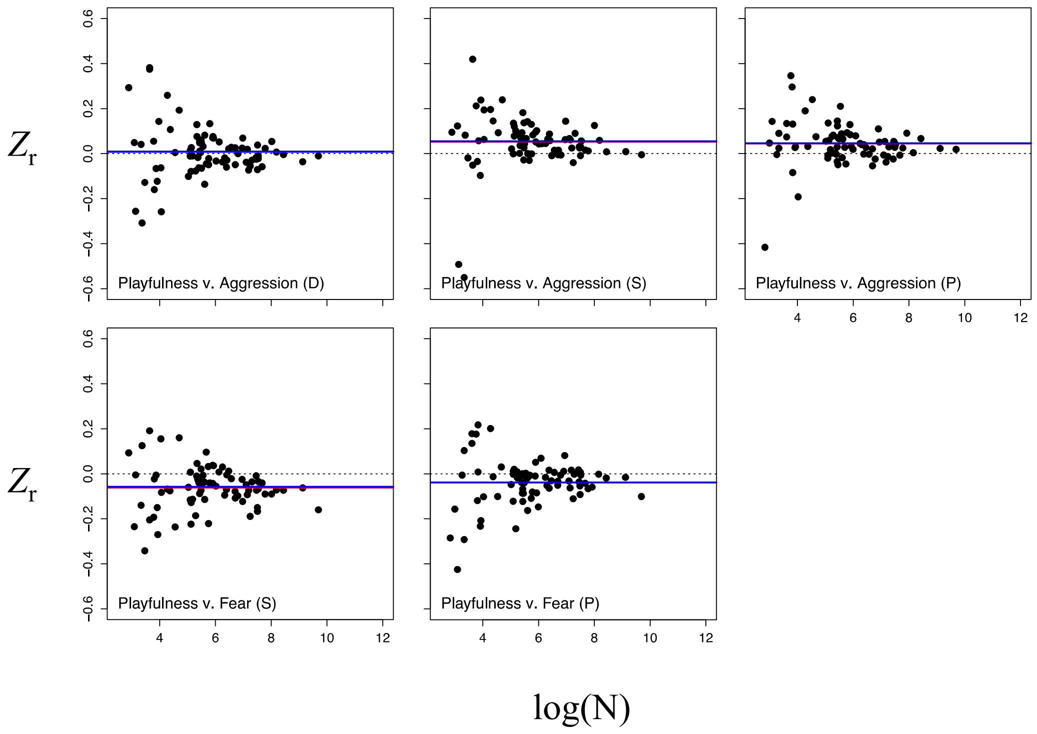

Supplementary Figure 4. Funnel plots for fearfulness correlations. Funnel plots for the correlations between two measurements of fearfulness and three measurements of aggression for all 78 dog breeds. The behavioural categories are Fearfulness - sudden threat (Fear (S)), Fearfulness - persistent threat (Fear (P)), Aggression - distant threat (Aggression (D)), Aggression - sudden threat (Aggression (S)), and Aggression-persistent threat (Aggression (P)). Estimated mean (red line) for a given correlation overlaps completely with the fitted regression mean (blue line). N = number of dogs within each of the 78 breeds in our study. Source data are available as a Source Data file.

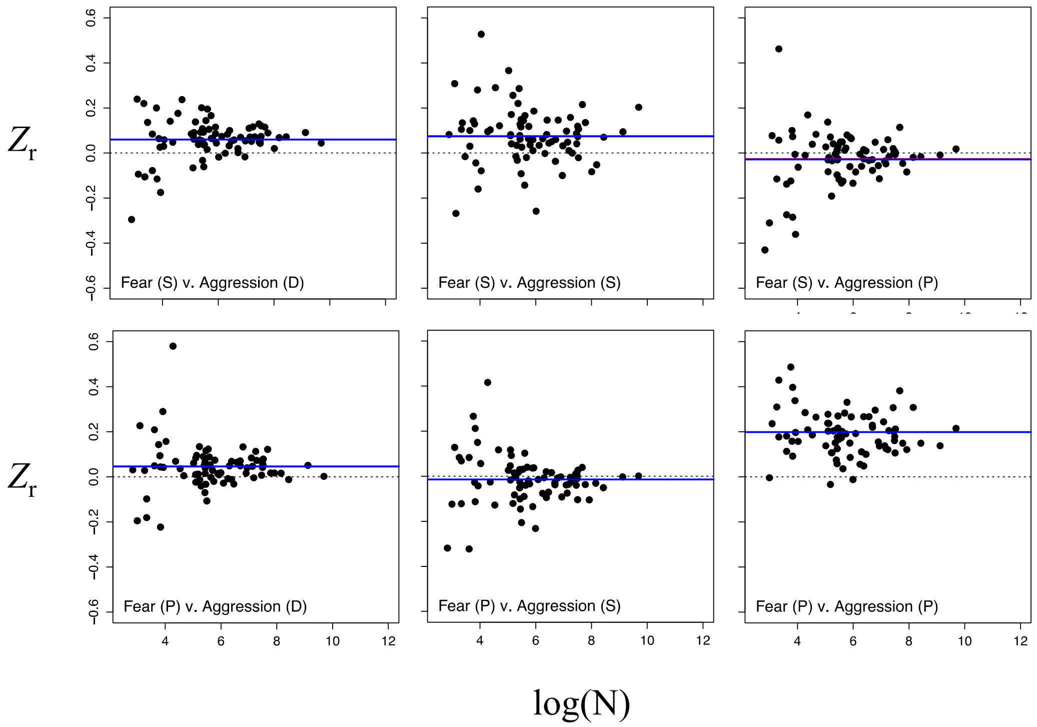

Supplementary References

1. Svartberg, K. & Forkman, B. Personality traits in the domestic dog (*Canis familiaris*). *Applied Animal Behaviour Science* **79,** 133–155 (2002).
