# Supplementary material for: Behavioural correlations of the domestication syndrome are decoupled in modern dog breeds
Source: Nat Commun. 2019 Jun 3;10:2422. doi: 10.1038/s41467-019-10426-3 (PMC6546797; doi:10.1038/s41467-019-10426-3)
Supplement: Supplementary file 3 — Reporting Summary [file 41467_2019_10426_MOESM3_ESM.pdf]

nature research

Corresponding author(s): Christina Hansen Wheat

Last updated by author(s): Apr 26, 2019

Reporting Summary

Nature Research wishes to improve the reproducibility of the work that we publish. This form provides structure for consistency and transparency in reporting. For further information on Nature Research policies, see Authors & Referees and the Editorial Policy Checklist.

Please do not complete any field with "not applicable" or n/a. Refer to the help text for what text to use if an item is not relevant to your study.
For final submission: please carefully check your responses for accuracy; you will not be able to make changes later.

Statistics

For all statistical analyses, confirm that the following items are present in the figure legend, table legend, main text, or Methods section.

n/a	Confirmed	
☐	☒	The exact sample size (n) for each experimental group/condition, given as a discrete number and unit of measurement
☐	☒	A statement on whether measurements were taken from distinct samples or whether the same sample was measured repeatedly
☐	☒	The statistical test(s) used AND whether they are one- or two-sided<br>*Only common tests should be described solely by name; describe more complex techniques in the Methods section.*
☐	☒	A description of all covariates tested
☐	☒	A description of any assumptions or corrections, such as tests of normality and adjustment for multiple comparisons
☐	☒	A full description of the statistical parameters including central tendency (e.g. means) or other basic estimates (e.g. regression coefficient) AND variation (e.g. standard deviation) or associated estimates of uncertainty (e.g. confidence intervals)
☐	☒	For null hypothesis testing, the test statistic (e.g. F, t, r) with confidence intervals, effect sizes, degrees of freedom and P value noted<br>*Give P values as exact values whenever suitable.*
☐	☒	For Bayesian analysis, information on the choice of priors and Markov chain Monte Carlo settings
☐	☒	For hierarchical and complex designs, identification of the appropriate level for tests and full reporting of outcomes
☐	☒	Estimates of effect sizes (e.g. Cohen's d, Pearson's r), indicating how they were calculated

Our web collection on statistics for biologists contains articles on many of the points above.

Software and code

Policy information about availability of computer code

Data collection	The Swedish Kennel Club
Data analysis	R Studio v.1.0.143, Mesquite v.3.51

For manuscripts utilizing custom algorithms or software that are central to the research but not yet described in published literature, software must be made available to editors/reviewers. We strongly encourage code deposition in a community repository (e.g. GitHub). See the Nature Research guidelines for submitting code & software for further information.

Data

Policy information about availability of data

All manuscripts must include a data availability statement. This statement should provide the following information, where applicable:

- Accession codes, unique identifiers, or web links for publicly available datasets
- A list of figures that have associated raw data
- A description of any restrictions on data availability

The data that supports the findings of this study are available from the Swedish Kennel Club but restrictions apply to the availability of these data, which were used under license for the current study, and so are not publicly available. Data are however available from the authors upon reasonable request and with permission of the Swedish Kennel Club.

Field-specific reporting

Please select the one below that is the best fit for your research. If you are not sure, read the appropriate sections before making your selection.

☐ Life sciences ☐ Behavioural & social sciences ☒ Ecological, evolutionary & environmental sciences

Ecological, evolutionary & environmental sciences study design

All studies must disclose on these points even when the disclosure is negative.

Study description	To achieve our main aim of assessing direction-specific differences in behavioural correlations between ancient and modern breeds, we first extracted residual values from behavioural test scores for all dogs, while accounting for potentially confounding variables. For each breed we then generated a correlation matrix based on these residual behavioural scores, with correlation coefficients (r) for each pairwise combination of behaviours. Using these correlations coefficients, and their associated sample sizes, we calculated Zr values using Fisher's transformation weighted by sample size and the sampling variances (Vz) for each behavioural correlation and breed. This approach allowed us to generate sample size-corrected effect sizes that indicate the strength and direction of associations between behaviours. We then tested if within-breed correlations predicted to be either positive or negative went in the direction predicted by the domestication syndrome hypothesis and if the strength of behavioural correlations differed between ancient and modern breeds by fitting the Zr and Vz values in a Bayesian phylogenetic meta-analysis that included breed and the type of behavioural correlation as random effects.

Research sample	The research sample consisted of 76,158 dogs representing 78 different registered dog breeds. Of the 78 dog breeds, seven belonged to the ancient breed group (N = 251) and 71 belonged to the modern breed group (N =75,907). All dogs were purebred and 1-4 years old. Males and females were equally represented in the research sample.
Sampling strategy	No sample size calculation was performed. The sample size was determined by the availability of dogs/breeds in the Swedish Kennel Club's database.
Data collection	This study was based on data obtained from the Swedish Kennel Club's database
Timing and spatial scale	The research sample was based on behavioural tests performed from 1997 - 2013
Data exclusions	No data was excluded
Reproducibility	No attempts to repeat the experiment has been made.
Randomization	Dog breeds were divided into Ancient and Modern breeds based on their detectable admixture with wolf.
Blinding	Data was collected by the Swedish Kennel Club previous to study conception. Data was therefore collected blind to the purpose of the study.

Did the study involve field work? ☐ Yes ☒ No

Reporting for specific materials, systems and methods

We require information from authors about some types of materials, experimental systems and methods used in many studies. Here, indicate whether each material, system or method listed is relevant to your study. If you are not sure if a list item applies to your research, read the appropriate section before selecting a response.

Materials & experimental systems

n/a	Involved in the study
☒	☐ Antibodies
☒	☐ Eukaryotic cell lines
☒	☐ Palaeontology
☐	☒ Animals and other organisms
☒	☐ Human research participants
☒	☐ Clinical data

Methods

n/a	Involved in the study
☒	☐ ChIP-seq
☒	☐ Flow cytometry
☒	☐ MRI-based neuroimaging

Animals and other organisms

Policy information about studies involving animals; ARRIVE guidelines recommended for reporting animal research

Laboratory animals	No laboratory animals were used. All animals were privately owned pets.
Wild animals	No wild animals were used. All animals were privately owned pets.
Field-collected samples	No samples were collected in the field. All animals were privately owned pets.
Ethics oversight	According to Swedish legislation, no ethical permission was needed for this study. All dogs were privately owned pets. Dog owners exclusively made the decision to enter their dogs in the DMA test procedure and could retract the dog from the test at any time during the DMA.

Note that full information on the approval of the study protocol must also be provided in the manuscript.
