# Supplementary material for: Behavioural correlations of the domestication syndrome are decoupled in modern dog breeds
Source: Nat Commun. 2019 Jun 3;10:2422. doi: 10.1038/s41467-019-10426-3 (PMC6546797; doi:10.1038/s41467-019-10426-3)
Supplement: Supplementary file 4 — Description of Additional Supplementary Files [file 41467_2019_10426_MOESM4_ESM.pdf]

**Description of Additional Supplementary Files**

File Name: Supplementary Data 1

Description: Sociability correlations. See separate data file. Zr values for the Pearson correlations (r) on residual values for behavioural scores across 78 breeds for (in separate tabs) correlations between Sociability (Soc) and Playfulness (Play), Aggression – persistent threat (AggrP), Aggression – sudden threat (AggrS), Aggression – distant threat (AggrD), Fearfulness – sudden threat (FearS), Fearfulness – persistent threat (FearP). Given is also Breed, Breed category (Cat, Ancient and Modern), expected correlation direction (Exp_cor), sampling variance (Vz) and sample size (n).

File Name: Supplementary Data 2

Description: Playfulness correlations. See separate data file. Zr values for the Pearson correlations (r) on residual values for behavioural scores across 78 breeds for (in separate tabs) correlations between Playfulness (Play) and Aggression – persistent threat (AggrP), Aggression – sudden threat (AggrS), Aggression – distant threat (AggrD), Fearfulness – sudden threat (FearS), Fearfulness – persistent threat (FearP). Given is also Breed, Breed category (Cat, Ancient and Modern), expected correlation direction (Exp_cor), sampling variance (Vz) and sample size (n).

File Name: Supplementary Data 3

Description: Fearfulness correlations. See separate data file. Zr values for the Pearson correlations (r) on residual values for behavioural scores across 78 breeds for (in separate tabs) correlations between two measures of Fearfulness: Fearfulness – sudden threat (FearS) and Fearfulness – persistent threat (FearP) and three measurements of Aggression: Aggression – persistent threat (AggrP), Aggression – sudden threat (AggrS), Aggression – distant threat (AggrD). Given is also Breed, Breed category (Cat, Ancient and Modern), expected correlation direction (Exp_cor), sampling variance (Vz) and sample size (n).
